# Shared realities, therapeutic settings, and countertransference in times of crisis: a multinational qualitative study of psychotherapists’ experiences

**DOI:** 10.1186/s40359-025-02962-z

**Published:** 2025-06-11

**Authors:** Rose Marie Nassif, Rahmeth Radjack, Nour Beaini, Tonnie Choueiri, Gabriel Binkowski, Elisabetta Dozio, Mathilde Laroche Joubert, Marie-Rose Moro, Layla Tarazi-Sahab, Mayssa’ El Husseini

**Affiliations:** 1 Université Paris Sorbonne Nord, UTRPP, Villetaneuse, 93430 France; 2 Université Paris-Saclay, UVSQ, CESP, Team DevPsy, Villejuif, Inserm 94807 France; 3 APHP, Hôpital Cochin, Paris, Maison de Solenn, 75014 France; 4 Department of Psychology, Saint-Joseph University, Beirut, Lebanon; 5 Laboratory of Psychoanalysis, Society and Politics at the, University of Sao Paulo, São Paulo, Brazil; 6 CLIPSYD, A2P, Université Paris Nanterre, Nanterre, 92000 France; 7 Université Paris Cité, PCPP, Boulogne-Billancourt, 92100 France; 8 MCU Université de Picardie Jules Verne, CHSSC EA 4289, Amiens, 80000 France

**Keywords:** Covid 19, Setting, Meta-frame, Trauma, Crisis, Countertransference, Qualitative research

## Abstract

**Objectives:**

The main aim of this study is to capture the impact of shared external realities — such as global crises — on the therapeutic process, with particular attention to how these events challenge the traditional structures of psychoanalytic and psychotherapeutic work. More specifically, the study seeks to understand how mental health professionals perceive and experience these disruptions, how they have been compelled to question their clinical frameworks, and how they have adapted their practices to maintain therapeutic continuity in a context marked by uncertainty, instability, and the frequent need for improvisation.

**Methods:**

We conducted a qualitative study among a population consisting of mental health professionals, which were recruited in multiple countries through associations and societies of psychologists, psychotherapists, and psychoanalysts. Data was collected using semi-structured individual interviews. A total of 40 participants were interviewed. The interviews were analyzed using interpretative phenomenological analysis (IPA), which allowed for a dynamic exploration of the participants' lived experiences.

**Results:**

The analysis of the data revealed three superordinate themes that are best presented by following the journey of the mental health professional as he or she navigates from external reality (1) to virtual reality (remote therapy) (2), ultimately plunging into the internal reality (internal reorganization linked to his or her ability to create novelty within these complex situations) (3).

**Conclusions:**

Our research shows that analysts and therapists experienced significant challenges to their professional identity due to external factors, including the COVID-19 pandemic. These shared experiences enabled the mobilization of creative internal resources and encouraged improvisation within an established framework. This facilitated working through the crisis and supported ongoing professional practice.

## Practitioner points

- Impact of External Reality: Recognize how global events, like the COVID-19 pandemic, can disrupt and transform professional identities and practices in psychotherapy.
- Navigating Virtual Realities: Learn strategies to transition from traditional in-person therapy to virtual platforms while maintaining therapeutic effectiveness.
- Ongoing Professional Development: Highlight the critical role of continuous professional development and adaptability in sustaining effective practice during challenging times.

## Introduction

### Contextual background

This collaborative research, born in response to the Covid-19 health crisis, brings together researchers from Lebanon, France, Italy, and Brazil. It explores how mental health professionals have had to question and adapt their practices and settings in order to continue working with their patients. Amidst this global pandemic, practitioners found themselves navigating unfamiliar territory marked by uncertainty and the need to improvise. This uncertainty stemmed from the sudden collapse of institutional, medical, and social frameworks that usually support clinical work, combined with heightened personal and collective anxieties. This challenge was further compounded in countries where prior conflicts have left populations grappling with their own anxieties, exacerbated by the convergence of global health issues and political situations, which lack a regulating third party. In such contexts, the pandemic was experienced not only as a health crisis but also as a multiplier of historical trauma and socio-political vulnerability.

### The invasion of the meta-frame

This study delves into the impact of the invasion of the “meta-frame” [1] in the clinical setting.

The meta-frame is a necessary condition for the functioning of the frame, representing a frame for the frame. Kaës initially introduced the meta dimension of the frame in 2012. He revisits various authors and psychoanalysts such as Bion, Pichon-Rivière, J. Bleger, Anzieu, and Gibelot and articulates the principles of the container and meta-container associated with the meta-frame. The container primarily functions to contain and transform elements of the psyche. A meta-container, on the other hand, serves as the contextual framework that enables and sustains the existence of a container. [1].

Various disruptions like pandemics, wars, natural disasters, revolutions, socio-political crises, and economic crises, among others, disturb external reality, causing changes that destabilize the meta-frame. Psychotherapists must adapt their theoretical knowledge, practices, and settings to these changes. When the meta-frame becomes unstable, psychotherapists depend on their therapeutic alliance with patients to continue their work. This bond encompasses both the therapists’ internal world and the shared interactive space cultivated during therapy [2]. Within this intermediate space, various emotions and objects emerge, supporting the therapeutic process. Even when disruptions occur in the meta-frame, this connection maintains the essential"containing shell and excitatory shield,"as described by Anzieu [3], which is vital for continuing therapeutic work.

### Countertransference and shared crises

A central concern in such situations is the therapist’s countertransference, defined by Laplanche and Pontalis [4] as “the entirety of the analyst’s unconscious reactions to the patient’s own transference.” In contemporary clinical usage, the term refers to both conscious and unconscious emotional responses, often shaped by the therapist’s own history, emotional sensitivities, and internal conflicts [5]. In crisis contexts, countertransference becomes more complex and layered, as therapists are not only witnesses but also participants in the same destabilizing reality.

Over the past decade, many studies have investigated the impact of trauma work on therapists who work with trauma patients. They show interesting subtleties in countertransference reactions to trauma narratives and shed light on processes indicative of trauma transmission [6–8].

Puget and Wender [9] introduced the concept of"superimposed worlds"to describe scenarios where traumatic events discussed in therapy sessions can distort and reshape the therapist’s capacity to listen and analyze. This situation raises questions about the asymmetry [10] between therapist and patient, especially when both are exposed to the same traumatic event. In such cases, boundaries in the therapeutic relationship become blurred. Kac Ohana [10] conceptualized this as a confrontation with *a world deprived of fantasy*—a psychic space where the capacity to symbolically process experience collapses.

### Socio-political dimensions of the crisis

The literature discusses various perspectives on the psychological and political impacts of the Covid-19 crisis. Bollas [11] describes the"over determination"of meanings during the crisis in the United States, highlighting factors like the pandemic, presidential actions, and social unrest, leading to a fractured societal reality. Rustin [12] emphasizes the significance of Bion's concept of the"container"in understanding the crisis. He notes the collapse of traditional structures of containment and its implications for public compliance with measures. In Brazil, Kupperman [13] examines President Bolsonaro's denialist statements regarding the pandemic, categorizing them into three forms: illusory, hypocritical, and pragmatic denialism, all of which have profound consequences on public health. The authors suggest that psychoanalytic insights can illuminate the complex interplay between political and psychological forces during the pandemic, underscoring the need for societal change.

### Review of literature

Several studies have examined how therapists worldwide adapted to the pandemic. Florio et al. [14] used Interpretative Phenomenological Analysis (IPA) to explore how Italian psychotherapists coped with altered therapeutic dynamics, identifying themes such as boundary management and professional identity challenges. Meneguzzo et al. [15] analyzed clinical narratives from practitioners and found that the pandemic prompted shifts in therapists'identity and empathy processes, revealing the importance of peer support and supervision. Aafjes-van Doorn et al. [16] highlighted early experiences of U.S. psychotherapists, including technological adaptation, blurred work-life boundaries, and emotional fatigue. Similarly, Filippou and Giannouli [17] found that Greek integrative psychotherapists were able to develop adaptive strategies in response to pandemic-related distress, drawing on the flexibility of their theoretical orientation to meet clients'changing needs. Tragantzopoulou et al. [18] further documented the emergence of psychological exhaustion and highlighted the role of supervision, peer support, and institutional frameworks in helping therapists sustain their professional functioning.

### The shift to remote therapy

One of the most visible changes during the pandemic was the sudden and widespread adoption of remote therapy. Before the pandemic, remote therapy had been a topic of academic interest but has since become a central concern in contemporary psychotherapy. Simpson and Reid [19] reviewed over two decades of research on therapeutic alliance, concluding that effective therapeutic relationships can be developed through video-based psychotherapy, comparable to in-person sessions. Similarly, Norwood et al. [20] conducted a systematic review and meta-analysis, confirming that videoconferencing psychotherapy leads to strong therapeutic alliances and positive outcomes. Bekes and Aafjes-van Doorn [16] emphasized that psychotherapists’ perceptions of online therapy are influenced by their prior experiences, including their therapeutic approach and familiarity with virtual settings. On the other hand, Mitchell's [21] review pointed out the lack of qualitative research examining therapists'subjective experiences and the deeper meanings associated with online therapy.

### Research aim

Our aim in this research is to understand how an external shared reality can alter the psychotherapist's working methods, listening capacity, and observations with patients, by exploring the impact of the invasion of the"meta-frame"[1] in the clinical setting. In this context, the study explores how therapeutic work can maintain continuity, adapt, and transform in response to shared disruptions and changing clinical realities.

## Materials and methods

This is an exploratory qualitative study approved by the research ethics board of CER U-Paris.

N° IRB: 00012020–65, the research ethics board of the Saint Joseph University in Beirut USJ-2020–214 and the national ethics committee of the University of São Paulo CAAE: 46,322,821.7.0000.5561.

### Theoretical framework

Psychoanalysis serves as a key theoretical backdrop for this study. Drawing from psychoanalytic concepts such as the meta-frame, trauma and countertransference, the research focuses on how therapists process and adapt to the disruption caused by an external shared reality like the COVID-19 pandemic. These concepts not only informed the interview design but also guided the interpretation of the data.

### Methodology

This study employed Interpretative Phenomenological Analysis (IPA) [22], a qualitative approach rooted in phenomenology and hermeneutics. IPA involves a'double hermeneutic'process, where researchers analyze how participants make sense of their own lived experiences, acknowledging the inherently subjective and interpretive nature of understanding. Guided by phenomenological principles, IPA delves deeply into individuals’ experiences to uncover the essence of their subjective realities. A key feature of IPA is its idiographic focus, which prioritizes the unique and personal nature of each participant's experience rather than striving for broad generalizations. Through an iterative and systematic process of data collection and analysis, IPA allows researchers to explore the intricate meanings people ascribe to their experiences, offering insights into the complexities of perception and interpretation within particular contexts.

As a research team, we developed an interview guide aimed at thoroughly exploring the objectives of this study. This guide was translated into several languages—including English, Italian, and Portuguese—to facilitate interviews with participants from different countries: Brazil, Italy, France, Lebanon, and the United States. The guide was gradually refined during the study to reflect new insights and emerging themes. Table 1 describes in detail the the thematic areas explored in the interview [23].

**Table 1:** Interview guide

I—Impact of the external situation (Covid/confinement) on the participants
1- Could you tell me how you experienced the news of the pandemic and the lockdown?
2- With what images would you describe the lockdown?
3- What did you observe in yourself as emotions, behaviors, physical changes?
4- What impact did this have on your creative abilities?
5- Did this situation bring back memories of similar experiences?
II- Impact of the situation on participants’ work with clients
6- What decisions did you take to ensure continuity of work?
7- How did you put in place virtual/distance work?
8- What impact did online work have on you? On your patients?
9- What were your habits concerning clients? How did these habits change with online work?
11- How did your ability to adapt and to transform things creatively come into play during the lockdown?
12- Did you notice changes in your way of being? of doing? (posture, ways of interacting, communicating, relational strategies)
13- How was the notion of intimacy affected?
14- Was there an invasion by patients into the private space? Did you observe their environment more intensely?
15- What difficulties do you meet with online work with patients?
16- With the changes to your life rhythm, was the time allotted to sessions respected? (slowness, change of time, technical and material issues)
17- How did the notion of debt come into play? (think of link with payment modalities)
18- What impact did the absence of the body of your patient in the workspace have on you? What impact on the patient?
19- What are the challenges of working in this situation? What are the advantages of working online or remotely?
20- How did you maintain the connection and interaction with your colleagues?
III- Impact of the situation on countertransference
21- Could you tell me about your dreams? The patients’ dreams?
22- What images come to you while listening to your patients?
23- Could you describe more specifically the unfolding of a course of therapy that marked you?
24- Could you tell me what you experienced (feelings, thoughts, physical reactions) when a patient would tackle the subject of confinement, of the disease? (Distinguish if this is linked to previous context of political instability, economic crisis etc.)
25- In what ways could you relate to some of the patients’ experience?
26- How did you feel about your internal availability and your ability to listen?
27- What are your thoughts about potential traumatic resurgences or trauma traces in patients after the pandemic and the confinement?
28- What do you think about traumatic resurgences or potential trauma traces in yourself after the pandemic and the confinement?
29- Do you think that new elements will emerge in your relationship with your patients after this situation?
IV- After the confinement?
30- How do you imagine the end of the confinement? (what pictures come to mind, ideas, thoughts)
31- How do you imagine going back to work after the confinement? What difficulties could you encounter with your patients?
32- Would you be more open to accept that patients do their therapies mostly or exclusively online?
Theme V was added to the interview of the Lebanese participants
V- Impact of Beirut explosion
33- Could you tell us about your situation at the clinic at the moment of the explosion on the 4th of August? *(clinical situation)*
34- Could you describe the damages and possible injuries that resulted after the explosion?
35- When and how did you go back to clinical practice after this event?
36- How would you describe your feelings, your thoughts and your experience during the period that followed the explosion of the 4th of August?

Due to the pandemic, most of the interviews were conducted online. Each interview lasted approximately 1.5 h on average. They were recorded, transcribed, anonymized, and then translated into French for analysis. Participants were informed of the study's goals through an information letter and introductory meeting. Oral and written consent were obtained from all participants prior to their involvement.

### Participants

The inclusion criteria for this study were professionals in the field of mental health, specifically clinical psychologists, psychiatrists, psychotherapists, or psychoanalysts. These professionals were required to be actively practicing in either private practice or institutional settings. Additionally, they should have maintained ongoing treatment or follow-up with adult and/or child patients throughout the period of confinement.

The recruitment of participants took place in the different countries of the researchers involved: France, Lebanon, Italy, Brazil and USA through associations and societies of psychologists, psychotherapists and psychoanalysts.

In all 40 participants were interviewed. Participants’ characteristics are described in Table 2.

**Table 2:** Characteristics of participants

Participant	Sex	Age	Country of origin	Country of practice	Profession/method	Population
1	F	64	Lebanon	Lebanon	Clinical psychologist/psychoanalysis	Adults/teens/children
2	M	44	Lebanon	Lebanon/France	Clinical psychologist/CBT	Adults/couples
3	F	34	Lebanon	Lebanon	Clinical psychologist/psychoanalysis	Adults
4	M	44	Lebanon	Lebanon	Clinical psychologist/EMDR/NLP	Adults/teens/children
5	F	43	Lebanon	Lebanon	Clinical psychologist/Psychodynamic therapy	Adults/teens/children
6	F	44	Lebanon	Lebanon	Clinical psychologist/psychoanalysis	Adults
7	F	49	Lebanon	Lebanon	Clinical psychologist/psychoanalysis	Adults/teens/children
8	F	38	Lebanon	Lebanon	Clinical psychologist/Psychodynamic therapy	Adults/teens
9	F	42	Lebanon	Lebanon	Clinical psychologist/psychoanalysis	Adults/teens/children
10	M	63	Lebanon	Lebanon	Clinical psychologist/psychoanalysis	Adults
11	F	34	Lebanon	Lebanon	Clinical psychologist/Psycho-organic therapy	Adults
12	F	35	Lebanon	Lebanon	Clinical psychologist/psychoanalysis	Adults
13	F	40	Lebanon	Lebanon	Clinical psychologist/Analytic therapy	Adults
14	F	34	Lebanon	Lebanon	Clinical psychologist/CBT	Adults/teens/children
15	M	43	Lebanon	Lebanon	Clinical psychologist/psychoanalysis	Adults
16	F	54	France	France	Psychiatrist/psychotherapist/anthropologist	Adults
17	F	38	France	France	Clinical psychologist/Analytic therapy	Adults/teens/children
18	F	49	France	France	Psychiatrist/psychoanalyst	Adults/teens/children
19	F	47	France	France	Clinical psychologist/Analytic therapy	Adults/groups
20	F	70	France	France	Psychiatrist/psychoanalysis	Adults/groups
21	F	44	France/Lebanon	France	Psychologist/gestalt therapy/Music therapy	Adults
22	F	39	Brazil	Brazil	Clinical psychologist/psychoanalysis	Adults/teens/children
23	F	69	Brazil	Brazil	Psychologist/psychoanalysis	Adults
24	F	28	Brazil	Brazil	Psychologist/psychoanalysis	Adults/children
25	M	30	Brazil	Brazil	Clinical psychologist/psychoanalysis	Adults
26	F	36	Brazil	Brazil	Psychoanalysis	Adults
27	F	33	Brazil	Brazil	Clinical psychologist/psychoanalysis	Adults/couples/families
28	F	48	Brazil	Brazil	Gestalt therapy	Teens/children
29	F	63	Brazil	Brazil	Psychologist/psychoanalysis	Adults/women victims of violence
30	F	45	USA/lebanon	USA	Psychiatrist/psychoanalysis	Adults
31	M	46	USA/Lebanon	USA	Psychiatrist/psychoanalysis	Adults
32	F	42	Portugal	Portugal	Clinical psychologist/psychoanalysis	Teens/young adults
33	F	31	Italy	Italy	Psychologist/systemic therapy	Adults/families
34	F	63	Italy	Italy	Psychologist/psychodynamic therapy	Adults/teens
35	M	43	Italy	Italy	Psychologist/systemic relational therapy	Adults/couples/families
36	F	41	Italy	Italy	Psychologist/analytical therapy/EMDR/sensorimotor therapy	Children/families
37	F	74	Italy	Italy	Psychoanalysis	Adults/groups
38	M	67	Italy	Italy	Psychiatrist/psychodynamic therapy	Adults/families
39	F	69	Italy	Italy	Psychoanalytical therapy	Adults/groups
40	F	61	Italy	Italy	Psychologist/evolutionary psychology	Children/adults/families

*CBT* cognitive behavioral therapy, *EMDR* Eye mouvement desensibilisation and reprocessing, *NLP* Neuro-linguistics programming

### Data analysis

The analysis adhered to a systematic approach guided by the IPA methodology [22]. Initially, all interviews were carefully transcribed and analyzed individually. To maintain consistency, the authors cross-checked their findings at various stages of the process. Reflecting the iterative nature of IPA, the analysis emphasized a dynamic engagement between the researcher and the textual data. Following transcription, the researchers repeatedly read through the text to deeply engage with the participants'narratives. This involved summarizing each interview and making annotations to capture interpretations, insights, and patterns within the data. Subsequent coding led to the identification of subordinate themes that captured key elements of the participants’ experiences At this interpretative stage, excerpts (verbatim) were selected to illustrate each theme. These themes were then grouped by content to develop broader superordinate themes that represented the shared meanings across the subordinate themes. To enhance the credibility of the study, three researchers independently analyzed the interviews, and any differences in interpretation were resolved through team discussions.

## Results

The analysis of the data revealed three superordinate themes that are best understood by tracing the journey of the mental health professional as he or she navigates from external reality to virtual reality (i.e., remote therapy), ultimately plunging into the internal realiy (namely, the internal reorganization linked to his or her ability to create novelty within these complex situations). Each superordinate theme came with constituent subordinate themes which are further elaborated in the following section (see Table 3).

**Table 3:** Overview of superordinate themes and subordinate themes

Superordinate themes	Subordinate themes
1. External Reality	1.1 The Context and Its Impact
	1.2 Traumatic Resurgences
	1.3 Resonances and Shared Trauma
2. Virtual Reality	2.1 The Notion of Space and Time
	2.2 The Sensory Aspect
	2.3 A Flexible Framework
3. Internal Reality	3.1 Restoring the Internal Framework
	3.2 Resorting to Theory
	3.3 Creativity and Improvisation

### External reality

#### The context and its impact

The profound impact of external realities, notably the multiple crises in Lebanon, the political crisis in Brazil, the *Black Lives Matter* movement in the United States and the terrorist attacks in France are the most salient events in the interviews analyzed.

The Lebanese context is characterized by a series of crises including economic collapse, the COVID-19 pandemic, and the Port of Beirut blast. The participants expressed how the external reality could at any moment break into the setting.

“*There are not only attacks from within, this year we had attacks from outside, which you can neither decide nor control, whether it's the virus, or socio-political and economic worries, and then the explosion.” P3.*

In the U.S., participants pointed to the convergence of the *Black Lives Matter* movement and the pandemic. As well as the varying security measures from state to state. The unprecedented diversity of responses at school, sector, community and even country level was also noted.

*“The whole Black Lives Matter thing, which in the U.S. coincided to some extent with all the dramas, and all the Covid crises. Exactly, because there were already security measures in our state that were really very different from other measures taken in other states in the United States, which were also generally very different from measures taken in my native country.” P30.*

The assassination of Samuel Paty in France has given rise to a great deal of fear and discussion, says a participant who works in a reception center for young migrants. He spoke of the fear of the team, of the confusion associating immigration with terrorism, and the worry that young people might become infected because of the stressful conditions of the pandemic.

*“The assassination of Samuel Paty, people spoke to me a lot, you know! They were marked, you know, very frightened, they were frightened, like us, first of all, the same little things we were, saying that they found it terrible and then, the fear of the amalgam if you like, migration, migration-terrorism.” P16.*

In Italy, participants felt that the culture of psychological well-being was lagging, and that the recovery plan did not address psychological issues. Work overload was a problem specific to the Italian context, with individuals grappling with the emergency situation and lacking time to manage their emotions. One Italian participant shared the disturbing experience of corpses in black bags and the pressure on resources at Alzano Lombardo.

*“My aunt remembers being awakened several times by the sound of zippers. They were put in the black bags—the corpses. In the same room where there were others, they weren't taken away. All these things were tiring, and Alzano Lombardo was like a lazaret.” P35.*

In Brazil, the government's trivialization of the situation, jokes about wearing masks and promotion of chloroquine were met with disappointment and disgust. Brazilian participants expressed a sense of powerlessness and abandonment, as politics interfered with care without logic or clear strategies. The aggravating factor of the government's management of the pandemic was seen as making the situation even more tragic in Brazil.

*“I was very disappointed, when you see the atrocities of the president of the republic, it's revolting, we needed shelter, we are in a total helplessness, abandoned children, when politics interferes in care without any logic or strategies.” P27.*

#### Traumatic resurgences

Participants' discourse also reveal the resurgence of past traumatic experiences, in various historical contexts such as wars, terrorist attacks and transgenerational trauma. In France, one participant reflected on the family repercussions of the Algerian war, highlighting how the current crisis has revived memories of their parents' experiences as Algerian repatriates. *"My family history comes back to me, my parents experienced the Algerian war head-on, they were part of what we call the Algerian repatriates". P19.*

In Lebanon, reflections on wartime shelters and the confinement of families during past events highlighted the parallel confinement experienced during the pandemic.

*"Certainly, the shelters, I was going to say, like earlier in the pictures, certainly the shelters when there were the events of the war, and the whole family also found themselves like that, confined or again in greater proximity in the shelters."P7.*

In Italy, a participant described the unsettling effect of pandemic-related imagery resembling war scenes, evoking comparable trauma. The fear of being emotionally blocked and the struggle to seek help drew parallels with past life experiences, highlighting the complex interplay between current challenges and past trauma for another participant.

*“It struck me, but the ambulances, the images and the news, as well as the processions of coffins, had the effect of a trigger and a similar trauma.” P36*

#### Resonances and shared trauma

Resonances and traumas shared with patients are also evoked. The heightened sensitivity to patients'traumas was analogously likened to a tuning fork, revealing a deep resonance with the emotional struggles of those they cared for. *"I was hyper-sensitive to my patients'traumas, really, you know, like a tuning fork.” P18.*

One participant demonstrated a deep awareness of the anxiety associated with the fear of death, illustrating an emotional terrain shared with his patients. *"I really became aware of the anguish linked to the fear of death". P19.*

Another participant described the moments of silence during remote sessions as a tsunami, connecting them to his personal family history, including the heavy burden of the Holocaust. This reflection highlights the profound impact of shared collective and personal traumas on the psychotherapist's experience, as well as on his or her ability to empathize with patients.

*“When she was silent in front of Skype, I said to myself: ah there's something going on, in fact, there's this very tsunamic silence; and in fact afterwards there were things coming, there's a sort of, you know, rumbling and then I think of things, I think of the tsunami in fact. My grandfather was Jewish, so there were deportations, so my great-grandfather died in the Auschwitz camps. It's a very heavy burden. So yes, this sort of silence before the tsunami, it also makes me think of the bombing, it also makes me think of war, well!” P18.*

### Virtual reality

The psychotherapist ensures the continuity of the therapeutic process by introducing the new device, the setting for remote therapy, the virtual framework, while the patient responds to the call by connecting, thus contributing to a co-creation that makes the encounter possible, for example patients who connect from their car or sitting in a park, unable to find a suitable space at home.

For most of the participants, the mandatory confinement and the transition to online therapy represented a novelty that gave rise to many anxieties, questions, and apprehensions. At the same time, this transition was seen as a relief, as it offered the possibility of continuing their professional activity and offering continuity to their patients.

We note several points in their comments about their experiences with the new virtual setting.

#### The notion of space and time

Particularly in relation to the spatial and temporal dimensions relating to sessions, the absence of transitional spaces between home and practice, which are traditionally spaces for reflection and preparation, was noted, altering the pre- and post-session ritual. *"All that time preparing for the session beforehand, all those sort of interstices between the home and the office that are spaces for elaboration, dreams and fantasies, were missing."P20.*

They describe a "collusion" between an intimate place and the world at large. *"A kind of collusion between a place that's very intimate, that's the place of your reflection, of your elaboration, all of a sudden, it was connected to the whole world."P16.*

This change is further accentuated by the compressed temporality of remote sessions: fewer silences, more responsiveness on the part of the therapist, but also more disinhibition on the part of patients, who reveal traumatic or phantasmatic content more rapidly, prompting one participant to speak of a "therapeutic accelerator pedal" during the initial phase of the pandemic. *“It's absolutely invaluable. And I was amazed at the efficiency and … I even had the perception, especially in the first part of the pandemic, that it was a therapeutic gas pedal. “P37.*

The newfound immediacy was underlined by the absence of traditional transitions between activities, with patients moving seamlessly from university classes to therapy sessions, blurring the boundaries between professional, academic and personal spheres.

#### The sensory aspect

The sensory aspect of online therapy was also noted. The absence of the body, as one participant noted, poses unique challenges, prompting therapists to find other ways to convey comfort and support through explicit verbal articulation and voice modulation. *“When you're in the presence of someone, it's easy to remain silent, the patient is thinking, I'm thinking, we're in this somewhat tranquil atmosphere, at a distance there's more tension to talk. It's as if they could take their thoughts a little further, I'd say, because we're at a distance.” P32.*

The fatigue associated with prolonged online sessions is a shared experience, with one participant expressing the taxing nature of virtual interactions, attributing it to compensation through the auditory dimension for the absence of the body. *“It was an enormous expenditure of energy. While the activity in front of me tires me, but also regenerates me, there's a cycle… there was just fatigue, so I felt my head tired, my eyes tired, my body tired… A great effort. “P35.*

The emphasis on the voice is clear: it is eroticized, felt to be too close. *“The voice was very present, so it was as if it penetrated the body, this omnipresence, that's the difference with therapies or analyses in person, because in person, there are attitudes, there's body language, so it's as if it absorbs the word a little more, or the voice.” P1.*

The visual component introduces complexities too, as participants are confronted with the constant visibility of their own image on the screen. This new self-awareness leads to discomfort. Access to the patient's intimacy is also noted (patients in their beds, barefoot, their children and spouses seen on the screen, etc.). One participant felt that he was being offered more than he asked for. *"I had the impression of being seen more closely, which makes the patient when, when he has my image on a screen, he can be distracted by the fact that if he spends his time looking at my expressions, my wrinkles, my pimples, my all that and my make-up, he has a perception overload and that it's not necessarily useful to maintain"P20.*

For psychoanalysts, it was seen best to hold sessions over the phone.

*“The impact is very big, to see myself in the frame on the screen. In the little frame I think seeing yourself is something because the judgement is bigger when you see yourself.” P 27.*

#### A flexible framework

Another crucial aspect of the virtual framework concerns its flexibility or elasticity, as some participants pointed out. While many wished to maintain a certain constancy in session schedules, flexibility was envisaged differently when it came to financial arrangements. Therapists and psychoanalysts found themselves obliged to share their bank details to facilitate payments by bank transfer, an uncommon practice in the psychoanalytic field for our participants in Lebanon, France and Brazil. *"Now there are those who were really very, very afraid to leave their homes, so there weren't many of them, and I could make the exception that they could make a money transfer.” P3.*

The question of self-disclosure was also raised, as was that of the hybrid therapy after returning to face-to-face consultations—what justifies or would constitute sufficient reason to accept an online session, etc.?

*"Somewhere along the line, I revealed a little of the fear I have regarding the Covid issue, regarding the issue of this social distancing."P17"It's not usual to have two face-to-face sessions and one by phone, it's going to have effects…", and in my head I said to myself it's not usual but there's nothing usual anyway."P18.*

*“Resistance to come to the session and asking for a remote session Normally I accept, but when I see that there's resistance or things that need to be talked about why they don't come normally I address that “P32.*

Our results clearly indicate a disparity in the adoption of the virtual framework between psychoanalysts and therapists of other approaches, the latter generally showing themselves to be more comfortable with the flexibility and adaptations induced by the transition to online work.

*“I've always understood that in psychoanalysis you have to be open to the unexpected, to the new, to the different, to situations that are just right, to delicate situations, and I was very surprised by the psychoanalysts around me, how they came to be so perplexed, as I said, it seems that these people have never seen a psychotic, a child in whom these situations appear more often who very easily break the rules of the framework, so it was strange for me to realise how difficult it is for us, or for psychoanalysts, to have to deal with a totally unpredictable situation. I've always understood that the unexpected is part of our job.” P23.*

### Internal reality

The improvised framework, in these circumstances, requires creative rethinking of one's practice and the co-construction of a malleable framework that preserves the therapeutic process. To achieve this, it is essential to be able to restore one's internal framework, which requires both personal work and elaboration with colleagues.

#### Restoring the internal framework

Participants stressed the importance of returning to personal analysis, supervision, participation in writing groups and sharing with colleagues.

*"I went back into analysis of course after that, because it was impossible to keep feeling that the glass was going to come crashing down on me. And it was during this period that I was talking almost more than the patient himself."P9.*

*"So I was lucky to have participated in a writing group very early on."P7*

Some participants regained a sense of centeredness by getting involved in psychological support projects for the community. This was seen as a way of regaining energy by helping others, offering a source of mutual support. Nature walks, daily movement and bodily activities became essential elements of self-care, serving as a dynamic counterbalance to the intense activation experienced during the crisis.* “Daily walking, movement, the body in motion. I could figure out how to do the activity, in short, at that point I couldn't bear to stand still.” P21.*

#### Resorting to theory

The use of theory becomes a means of navigating through the complexity of online work, offering conceptual and practical guidance to preserve therapists'internal framework. This demonstrates their commitment to maintaining a solid theoretical foundation while adapting to the new demands of clinical practice.

*"I was more'Winnicottian'during the pandemic, and during all that's going on in Lebanon too, which can sometimes be worse than the pandemic. But I didn't lose sight of the analytical aspect of things. The analytical aspect in the sense of analytical technique."P10.*

*“I went to talk about it in supervision I went to say that I didn't have this rigidity this problem isn't a problem and we talked for a while about the meaning of doing a bank transfer, so I think it was also something that had already worried me because it was new, I had to understand what it would mean to the patient” P26.*

*"Sensorimotor therapy is wonderful. In the relationship with the person, I like it a lot, that is, everything is co-constructed with the patient, whereas in EMDR there's a condition, there's a hierarchical position. What helps me is to be able to recognize them and to listen to them a lot, i.e. I use sensorimotor training a lot to regulate myself, i.e. the therapist's regulation.” P37.*

#### Creativity and improvisation

Analysis of the results reveals how participants deployed creativity and improvisation in the context of online therapy during the pandemic. Practitioners adopted innovative approaches, including the creation of complex outdoor clinical environments to maintain care for vulnerable patients (migrants, women victims of violence etc.) underlining the need to adapt to new conditions. Virtual psycho-education projects on Covid were set up, integrating medical-health and psycho-emotional aspects in a comfortable way through interactive Zoom presentations. Certain participants extended the use of mindfulness practices in creative ways, even with initially hesitant patients, noting its positive impact on emotional stabilization. *"We talk about everything we've created with the children, and precisely how to, well, how to continue therapy, how to make teletherapy a possible creative nerve rather than something fetishistic". P18.*

Some participants also spoke of the feeling of being in a bubble during sessions, enabling them to think and elaborate with patients despite the chaos outside. *“Children get right into your belly and bring with them behaviors and content that also force you to create. To be flexible. So, for me, this period was a change of scene. “P36.*

Creativity was seen as a binding and libidinal element, offering a contrast with collective trauma, which is often associated with disintegration. Psychoanalysts stressed the need to be open to unexpected and delicate situations, recognizing that the pandemic brought unforeseen challenges that required creative solutions.

*"We were confronted with challenges that were really sources of creativity that we didn't suspect in ourselves, and it's all very well to say that psychoanalysis and psychoanalysts and all those interested in it are a little rigid, a little overtaken by time, by events *etc*. by the course of modern life *etc*., but we found that this was not the case."P15.*

## Discussion

This qualitative study aimed to explore psychotherapists'lived experiences when confronted with shared external realities with their patients. The analysis of therapists'interviews revealed a core aspect of their experiences: the feeling of insecurity stemming from the intrusion of external reality into their therapeutic setting and framework. Our results demonstrate the journey that this experience compelled psychotherapists to navigate, emphasizing the necessity of working through uncertainty in their practice.

"In a moment that affects the patient as much as the analyst, the analyst's anguish is decisive, especially if he risks being overwhelmed."says Bleger [24].

When the unexpected occurs from outside the session and breaks into the therapeutic framework, what remains of the communion between the two protagonists caught up in the same unexpected event, often with traumatic potential, depends on how the therapist works through it. In the case of our participants, our findings highlight the profound repercussions of the collective external reality, revealing the resurgence of past traumatic experiences. Furthermore, our study highlights the transmission of these traumas, resonating with patients and emphasizing the enduring impact of shared external challenges. Drawing on Ferenczi's model of countertransference [25], therapists navigate through three phases: In the first, the analyst is emotionally overwhelmed by the patient's feelings; in the second, there's an effort to control these reactions, risking detachment; and in the third, achieved through introspection and analysis, the analyst uses their own countertransference as a tool for therapeutic understanding, allowing both patient and analyst to explore previously unaddressed conflicts. The therapists involved in our study find themselves first oscillating between phases of uncertainty and disruption, questioning their professional identity; second and in a defensive phase resorting to theory, before allowing a phase of restoring internal framework and creative implications.

Our research aligns with existing studies examining the lived experiences of mental health professionals when faced with unique challenges in their clinical practice. These professionals are exposed to a spectrum of physical and mental health risks, such as secondary traumatization, burnout, anxiety, and depression. [26, 27] DiGiovanni et al. [28] examined the personal and professional effects of the COVID-19 pandemic on Child and Adolescent Psychiatrists in the USA, focusing on changes in development, practice, and values. Their findings highlighted a consensus among participants regarding the necessity for implementing change due to the pandemic. Sibeoni et al. [29] study involved Child and Adolescent Psychiatrists from 26 countries, their findings revealed three central axes of experience: disorganization of clinical practice in terms of lived time and space, disconcerting experiences with the body of both psychiatrists and patients, and predominant unpleasant emotions, particularly angst and loneliness, influencing their clinical practice. However, our research nuances these findings by indicating that despite the potentially destructuring and anxiety-provoking impact, we specifically reveal, that amidst these challenges, mental health professionals can undergo a transformative journey that fosters creativity, challenging the notion of mere disorganization and highlighting the potential for positive growth in the face of adversity.

Our participants acknowledged being overwhelmed by the intrusion of external realities into their environment, noting a loss of their capacity to contain and listen effectively. Some of them found returning to analysis or supervision, along with establishing a support network with peers, as strategies to regain their ability to engage with patients. In this, we agree with El Husseini in her research on countertransference in the trauma clinic [2], in which she demonstrates that personal analysis and supervision were a means for therapists to protect themselves from the impact of the trauma in their clinic, while others emphasized the need to benefit from them in order to transform what is deposited in the therapist's psyche. This result also addresses inquiries raised in Bekes et al.'s [16] study regarding the potential of peer support and supervision, during and after the pandemic, to enhance psychotherapists'reflective practice and consequently influence their therapeutic approach.

Changes and transformations in work practices (such as the switch to online therapy) challenge the notion of the permanence of the frame in its material and internalised dimension. The main protective and holding functions of the frame—for which therapists act as guarantors—are thus put to the test. The transition to online therapy, although a forced transition and a first experience for most participants, allowed them to continue working with their patients and even to take on new patients, which was a source of relief. However, it also raised concerns about the uncertainty surrounding the online setting and its impact on the therapeutic relationship with patients.

The findings clearly show a contrast in the acceptance of the virtual setting between psychoanalysts and therapists using alternative approaches. In general, the latter group is more comfortable with the flexibility and adaptations brought about by the move to online work.

Teleanalysis is currently a subject of significant controversy among psychoanalysts. Although, an increasing body of research supports the legitimacy and effectiveness of the technologically modified setting: Several authors [30–34] advocate for the merits of online psychoanalysis, arguing that comparable processes exist in both in-person and virtual analyses. Through vignettes or clinical cases, they reference the demonstration of transference and countertransference, unconscious communication, free association, free-floating attention, and emotional support, alongside interpretations.

We found in the literature that some psychoanalysts have reservations about teleanalysis which aligns with the results of our study. In the context of telephone sessions, various aspects of in-person interactions were notably absent, including physical presence, visual cues, and olfactory sensations. Although bodies are not physically present, they still exist in a muted capacity. Additionally, auditory perception becomes more pronounced, with voices penetrating the ear differently compared to in-person meetings [35]. Krzakowski [36] notes the fatigue experienced and the tendency of analysts to intervene more, he discusses the effects of remote sessions, particularly from its psychoanalytic ‘economic’ perspective he says"one of the paradoxes of teleconsultation is revealed both in a need to desexualize a relationship freed from its usual par-excitations, while the other side of the paradox lies in a need to re-sexualize a situation that can become too cold, at the risk of immobility". Patry [37] refers to the issues of lack and loss associated with the change of analytic setting in telephone sessions. The absence of the body deprives both patient and analyst of a "full transferential experience", she says. The analyst is content to rely solely on the analysand's words to "feed his reverie". Floating listening focuses on the voice, its intonation, and fluctuations. Can it remain floating, or becomes more vigilant?

This question of how analytic listening and presence are affected by remote settings leads naturally to a broader reflection on the analytic frame itself. De Staal [38] expresses the loss felt in moving from the couch to the screen, underlining the disappearance of a concrete material aspect deeply linked to the habitual working environment. This shift raises fundamental concerns: how can the setting be moved or rearranged without compromising the "ceremonial imposed during the sessions", as mentioned by Freud in 1913, and without destroying "the very edifice of psychoanalysis".

The discussion around the analytic frame is not new; it has been central to contemporary theorists who have sought to extend psychoanalysis beyond neurotic structures, such as Green, Donnet, and Roussillon. However, the current context of remote sessions introduces new challenges, making the issue even more pressing. The transition from the couch to the screen is not simply a matter of imitative transposition for fear of "doing anything". Instead, it requires a dose of inventiveness, a creative dimension accompanied by the recognition of losses and gains, thus fostering the"creation of the new"while preserving the initial"idealized model"[38]."When it comes to frameworks, the reference to ethics is quick to make its appearance… It sounds like a call to order, an injunction to"not do just anything". Do analysts lack reference points? Do they need to be supervised or framed? says Bleger [24].

In line with A. Green's [39] perspective, when a radical transformation of the framework is required, the analyst should turn to an internal framework, that resulting from his or her own internalized analysis. Analysts see their own psychic framework as the cornerstone of the analytic process, guaranteeing that they will take full responsibility for it.

The analytical process is internal, even if it is determined by stable and constant external conditions. The risk, therefore, lies not in the change of support (couch/screen) but rather in the more profound upheaval of our internal world in an external context that has become too unstable, too uncertain, which risks inhibiting our capacity for thought, reverie, and symbolization.

## Conclusion

The results of our research show that analysts and therapists have all gone through a period when their professional identity has been shaken and challenged by external factors. As these factors were shared at a national level, and some at an international level (Covid 19), colleagues were able to share their experiences and call on a group: the community of peers. The links forged through the crisis enabled the mobilization of creative internal resources, authorizing improvisation on the basis of an internal framework, but also based on a possible, authorized enunciation, opening up a sharing of the weight of the crisis.

## Data Availability

The data are not publicly available due to privacy restrictions. The raw de-identified data may be made available upon reasonable request from the corresponding authors.

## Abbreviations

IPA: Interpretative Phenomenological Analysis
CBT: Cognitive behavioral therapy
EMDR: Eye Movement Desensibilisation and Reprocessing
NLP: Neuro-linguistics programming
